# Iodine-131 labeled genistein as a potential radiotracer for breast cancer

**DOI:** 10.1016/j.heliyon.2020.e04780

**Published:** 2020-09-21

**Authors:** Danni Ramdhani, Eva Maria Widyasari, Maula Eka Sriyani, Quinzheilla Putri Arnanda, Hiroshi Watabe

**Affiliations:** a Department of Pharmaceutical Analysis and Medicinal Chemistry, Faculty of Pharmacy, Padjadjaran University, Sumedang, West Java 45363, Indonesia; b Applied Nuclear Science and Technology Center (PSTNT), National Nuclear Energy Agency of Indonesia (BATAN), Bandung, West Java 40116, Indonesia; c Division of Radiation Protection and Safety Control, Cyclotron and Radioisotope Center, Tohoku University, Sendai, Japan

**Keywords:** Pharmaceutical chemistry, Iodine-131, Genestein, Breast cancer, Radiotracer, Estrogen receptors beta

## Abstract

**Objective:**

Genistein is an isoflavone compound that has been proven to have anticancer activity and is capable of binding to estrogen β receptors with Selective Estrogen Receptor Modulators (SERMs) properties, and has a strong affinity to inhibit the development of cancer cells. This study is to determine the optimum conditions of the reaction in the synthesis process of compounds labeled ^131^I-genestein which can be potential for application of breast cancer diagnosis.

**Methods:**

Synthesis of ^131^I-Genistein compound labeling using the Chloramine-T iodination method. This method uses several parameter optimizations, including: pH conditions, the amount of chloramine-T oxidizer and sodium metabisulfite reducing agent. The radiochemical purity of the ^131^I-Genistein compound was determined using thin layer chromatography TLC-SG F_254_, and measured by SCA (Single Channel Analyzer). The radiochemical purity of labeled compounds must fulfill the requirements of the United States of Pharmacopeia.

**Results:**

Optimization of the synthesis conditions of the ^131^I-Genistein compound was obtained at pH 8, the amount of chloramine-T 0.225 mg, and the amount of Na-Metabisulfite 0.342 mg, with 30 min reaction time. This optimum condition produces radiochemical purity of 95.02 ± 0.76%.

**Conclusion:**

Products labeled ^131^I-Genistein meet radiochemical purity requirements according to USP requirements. The labeled compound is expected to be able to be used to detect breast cancer through a binding mechanism with estrogen receptors β.

## 1 Introduction

Breast cancer arises due to abnormal cell growth that duplicates uncontrollably and becomes malignant in the breast. There are many types of breast cancer, in general, ductal carcinoma in situ (DCIS) and invasive carcinoma. Other types, such as phyllodes and angiosarcoma tumors are very rare [1].

In the US in 2019, there will be an estimated 268,600 new cases of invasive breast cancer diagnosed in women; 2,670 cases diagnosed in men; and an additional 62,930 cases of in situ breast lesions ductal carcinoma in situ [DCIS] or lobular carcinoma in situ [LCIS] diagnosed in women, and invasive female breast cancer incidence rates increased slightly, by 0.4% per year [1]. In Indonesia, the highest incidence of cancer in women is occupied by breast cancer, which is 42.1 per 100,000 population with an average death rate of 1.7 per 1000 population in 2018 [2].

Expression approaches of estrogen receptors (ER), progesterone receptors (PgR), and HER2 receptors, and evaluation of clinical variables, such as nodal involvement, tumor size, histological type, tumor grade, and surgical margins, are widely used to determine treatment and also a prediction of effective breast cancer prognosis [3, 4].

Genistein [4′,5,7-trihydroxyisoflavone] (C_15_H_10_O_5_) is an isoflavone that is mostly found in soybeans. In nature, Genistein is in free form (aglycone) or glycosylated with O-β-D-glucoside derivative in position 7 and inactive form [5]. The results of in-silico testing using molecular docking method showed that genistein interacts with β-estrogen receptors, and strong bonds form and produce higher interaction stability [6]. In addition, the results of in-vitro studies indicate that Genistein selectively binds to ERβ with a strong affinity to inhibit the development of cancer cells [7].

Genistein also has been proven to have many pharmacological activities including inhibiting the work of the enzyme tyrosine kinase in preventing the development of breast cancer cells [8, 9]. Genistein can induce apoptosis in BRCA wild-type-1 and mutant BRCA cancer cells, induce apoptosis in breast cancer cells by reducing the level of expression of miR-155, micro-RNA, has the potential to inhibit Skp2 expression in breast cancer cells as an anticancer effect [10, 11, 12].

Iodine-131 has radioisotope emitting characteristics of γ and β particles, with the gamma energy specifications of 364 keV and β particles, and has a half-life of 8.1 days, and is widely used in applications in the field of nuclear medicine as radiotracers and radiotherapy [13]. I-131 can also be used for molecular imaging using SPECT because it produces γ-ray emissions during decay. Radiolabeling I-131 with molecules usually involves electrophilic aromatic substitution of phenolic or trialkylstannylated substrates using oxidizing agents (eg, Chloramine-T and iodogens); this usually produces high radiochemical yield [14]. Synthesis of ^131^I-Genistein labeled compound is intended as a compound for the diagnosis of breast cancer through specific reminder of estrogen receptors.

## 2 Materials and methods

Thin layer chromatography plate (TLC-SG F_254_) (Merck®), dose calibrator (Victoreen®), 5 μL micropipette, 10–100 μL, and 100–1000 μL (Eppendorf®), analytic balance (Mettler Toledo® Type AL 204), oven (Memmert®), Single Channel Analyzer (SCA) (ORTEC®), syringe (Terumo®), 10 mL glass vial.

Genistein (Sigma-Aldrich®), aquabides pro injection (IKA Pharma®), 0.1 N hydrochloric acid (Merck®), phosphate buffer 0.02 N pH 7.4, dimethyl sulfoxide (Merck), Whatman 1 paper, chloramine-T (Sigma-Aldrich®), chloroform (Merck®), methanol (Sigma-Aldrich®), sodium hydroxide 0.1 N; 0.5 N (Merck®), sodium metabisulfite (Merck®), radionuclide I-131 in the form of dilute Na^131^I, universal pH indicator (Merck®).

### 2.1 Optimization of pH

The optimum pH testing is done by varying the pH of 7, 8, and 9. 200 μl Genistein stock solution plus 30 μl chloramine-T solution. The pH is adjusted by adding 0.1N HCl or 0.1N NaOH. Then, the solution in the microcentrifuge tube is added 5–7 MBq/50 μl Na^131^I and incubated at room temperature while shaken with a mixer shaker for 30 min.

Then each microcentrifuge tube was added 60 μl Na_2_S_2_O_5_, and purification was carried out by liquid-liquid extraction using chloroform 3 times. The water phase and the chloroform phase are taken and then the activity is measured with a dose calibrator. Radiochemical purity in the aqueous phase was determined by thin layer chromatography and electrophoresis methods.

### 2.2 Optimization of chloramine-T and Na_2_S_2_O_5_ concentrations

Optimization of chloramine T concentration in the labeling technique with Iodine-131 is to make a difference in the amount of 10, 20, 30, 40, and 50 μl which will be added to every 5 microsentrifuge tubes that already contain 200 μl genistein solution. The two solutions are then shaken to dissolve completely. The pH is adjusted by adding NaOH 0.1 N and HCl 0.1 N to achive the optimum pH. Then, 50 μl of Na^131^I were added to each solution and incubated at room temperature and shaken with a mixer shaker for 30 min so that the mixture dissolved completely. Na_2_S_2_O_5_ solution with variations in the amount of 20, 40, 60, 80, and 100 μL was then added, and the shaking was done again. The final solution is then purified using the liquid-liquid extraction method using chloroform as much as 3 times.

The aqueous and chloroform phases are separated, and their radioactive activity is measured by dose calibrator. Radiochemical purity in the aqueous phase was determined by thin layer chromatography and electrophoresis methods. The optimum amount of chloramine-T is used in optimizing the amount of genistein ligands.

### 2.3 Percentage of radiochemical purity of compounds labeled ^131^I-Genistein

^131^I-genistein radiochemical purity measurements were carried out by thin layer chromatography using TLC-SG F254 and electrophoresis method. The radiochemical purity of ^131^I-Genistein is determined by the side products formed namely I_2_ and I^−^ which are impurities product. Determination of I_2_ impurities are carried out by the TLC method using the chloroform mobile phase. Whereas, for the measurement of the percentage of impurities I^−^ done by electrophoresis method using whatman paper no.1 with phosphate Buffer 0.2 N pH 7.4 as an electrolyte solution. Electrophoresis equipment installed with a voltage of 350 V in 60 min. The results of TLC-SG F254 and electrophoresis were measured by radioactive activity with SCA (Single Channel Analyzer).

The radiochemical purity of a labeled compound ^131^I-Genistein is calculated based on the percentage of I_2_ and I^−^ (impurity) using the following equation.

$$\text{\%}{\text{I}}_{2}=\frac{\text{Number of counts in top}}{\text{Total number of counts}}\times 100\text{\%}$$

$$\text{\%I}=\frac{\text{Number of counts in top}}{\text{Total number of counts}}\times 100\text{\%}$$

Calculation of labeled compounds ^131^I-genistein.

% ^131^I-Genistein = 100% - (% I_2_ +% I^−^) [15].

## 3 Results

### 3.1 Optimization of pH conditions

The physicochemical properties of Genistein is considered in the selection of pH optimization. Determination of pH 7, 8 and 9 in the iodine-131 labeling process with Genistein through pH adjustment with the addition of 0.1 N HCl solution or 0.1 N. NaOH, and The composition of the formula in determining the pH optimization is in Table 1.

**Table 1:** Formula for determining the pH labeling optimization of ^131^Iodine-genistein.

Genistein (10 mg/1mL)	Na^131^I (1.2 MBq/μL)	Chloramine-T (37.5 mg/5 mL)	pH	NaOH 0,1 N (μL)	HCl 0,1 N (μL)	Na_2_S_2_O_5_ (28.5 mg/5mL)
200 μL	50	30 μL	7	-	5	60 μL
200 μL	50	30 μL	8	-	-	60 μL
200 μL	50	30 μL	9	15	-	60 μL

**Figure 1:**
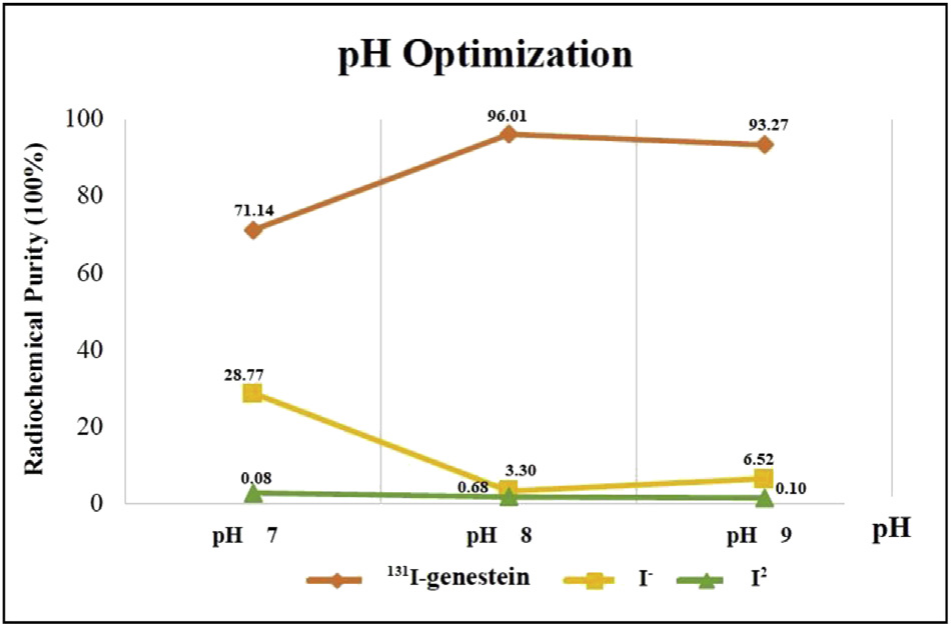
Results of pH optimization in the synthesis of compounds labeled ^131^I-Genistein.

The results of pH optimization in the synthesis of ^131^I-Genistein labeled compounds are in Table 2 and Figure 1. The results of pH optimization obtained pH 8, with radiochemical purity of 96.01% ± 0.62.

**Table 2:** The results of pH optimization on iodine-131-genistein labeling.

pH	Impurities Product		Radiochemical Purity (%)
	I_2_ (%)	I^−^(%)	
7	0.08 ± 0.02	28.77 ± 1.95	71.14 ± 1.97
8	0.68 ± 0.38	3.30 ± 0.23	96.01 ± 0.62
9	0.1 ± 0.013	6.52 ± 0.19	93.27 ± 0.20

### 3.2 Optimization of Chloramine-T and Na_2_S_2_O_5_ concentrations

The synthesis of labeled compounds ^131^I-Genistein using the chloramine-T method. The amount of chloramine-T determines the amount of oxidized iodine to bind to the genistein compound and determines the radiochemical purity of the formed compound. The chloramine-T concentration used was 37.5 mg/5 mL. In this study, the volume of chloramine-T varied from 10, 20, 30, 40 and 50 μL.

**Figure 2:**
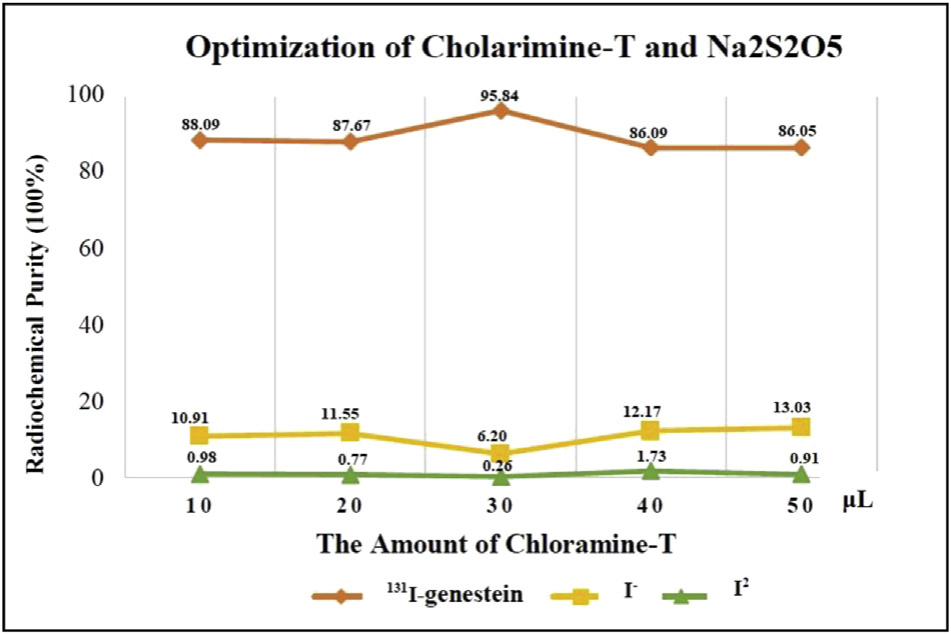
Results of Chloramine-T and Na_2_S_2_O_5_ optimization in the synthesis of compounds labeled ^131^I-Genistein.

Sodium metabisulfite solution was added immediately after the reaction time with a concentration of 2 times the concentration of chloramine-T. The concentration of sodium metabisulfite used in this study was 28.5 mg/5 mL. The volume of sodium metabisulfite varies to 20, 40, 60, 80, and 100 μL. The formula for determining the optimization of chloramine-T and Na_2_S_2_O_5,_ and the results of radiochemical purity are in Table 3 and Figure 2.

**Table 3:** The results of chloramine-T and Na_2_S_2_O_5_ optimization on ^131^I-Genistein labeling.

Genistein (μL)	Chloramine-T (μL)	Na_2_S_2_O_5_ (μL)	Impurities Product		Radiochemical Purity (%)
			I_2_ (%)	I^−^ (%)	
200	10	20	0.98 ± 0.24	10.91 ± 0.49	88.09 ± 0.73
200	20	40	0.77 ± 0.17	11.55 ± 0.85	87.67 ± 0.67
200	30	60	0.26 ± 0.05	6.20 ± 3.22	95.84 ± 3.27
200	40	80	1.73 ± 0.22	12.17 ± 0.91	86.09 ± 0.68
200	50	100	0.91 ± 0.19	13.03 ± 4.53	86.05 ± 4.73

The result of optimization of chloramine-T was 30 μL and sodium metabisulphite was 60 μL, with the obtained radiochemical purity of 95.84% ± 3.27.

## 4 Discussion

Optimization of the Genistein labeling process with Iodine-131 through the chloramine-T method under alkaline pH conditions. Genistein tends to be stable at neutral and basic pH. However, the stability of Genistein will decrease and be limited to strong base conditions [15]. Optimization results on the synthesis of compound ^131^I-Genistein were obtained at pH 8, with DMSO as a solvent. Genistein has high solubility in organic polar solvents such as ethanol, acetone, and DMSO but has lower solubility in water and dissolves under alkaline conditions [16, 17].

These results are also in line with the results of optimizing the labeling of genistein compounds with technetium-99m radioisotope with pH 8 as the optimum pH with purity reaching 91.97% ± 1.43% [18]. Optimization results at pH 8 indicate that this pH approaches physiological conditions, where the mechanism of action of genistein as an estrogen receptor antagonist will be stable, and can perform its function as an estrogen receptor antagonist beta sub unit [19].

The synthesis of compounds labeled ^131^I-Genistein using the chloramine-T method. The chloramine-T method is very widely used because it produces higher radiochemical purity compared to other methods [14]. Chloramine-T is a strong oxidizing agent which can oxidize radioactive iodine through radioiodination reaction by electrophilic substitution in the structure of genistein compounds to produce compounds labeled ^131^I-Genistein. The following is the reaction between chloramine-T and iodine:

CH_3_-C_6_H_4_SO_2_NaNCl + H_2_O → CH_3_-C_6_H_4_SO_2_NH_2_ + NaOCl

NaOCl + HI → HOI + NaCl [21].

Sodium metabisulfite is a reducing agent used to stop the labelling reaction by reducing I^+^ back to I^−^ so that there is no damage to the structure of genistein compounds and prevents the formation of many impurities I^−^ that is formed [20].

## 5 Conclusion

Synthesis of labeled compounds ^131^I-Genistein obtained optimum conditions including pH 8, the amount of chloramine-T 30 μL (37.5 mg/5 mL), the amount of Na_2_S_2_O_5_ 60 μL (28.5 mg/5 mL), the number of genistein ligands 200 μL (10 mg/1 mL), and 30 min reaction time with radiochemical purity of 95.02 ± 0.76%. These results meet the radiochemical purity requirements set by USP for labeled compounds more than 90%. The labeled compound of ^131^I-Genistein is expected to be used as a radiotracer for the diagnosis of breast cancer.

## Declarations

### Author contribution statement

Danni Ramdhani: Conceived and designed the experiments; Analyzed and interpreted the data; Wrote the paper.

Maula Eka Sriyani, Eva Maria W: Conceived and designed the experiments; Contributed reagents, materials, analysis tools or data.

Quinzheilla Putri A: Performed the experiments; Analyzed and interpreted the data.

Hiroshi Watabe: Wrote the paper.

### Funding statement

This research did not receive any specific grant from funding agencies in the public, commercial, or not-for-profit sectors.

### Competing interest statement

The authors declare no conflict of interest.

### Additional information

No additional information is available for this paper.

## References

[1] American Cancer Society (2017). Cancer Facts and Figures 2017. https://www.cancer.org/content/dam/cancer-org/research/cancer-facts-andstatistics/annual-cancer-facts-and-figures/2019/cancer-facts-and-figures-2019.pdf.

[2] Ministry of Health of the Republic of Indonesia (2019). Deteksi Dini Cegah Kanker. http://www.depkes.go.id/article/view/19020500001/deteksi-dini-cegah-kanker.html.

[3] Cheang M.C., Chia S.K., Voduc D., Gao D., Leung S., Snider J. (2009). Ki67 index, HER2 status, and prognosis of patients with luminal B breast cancer. J. Natl. Cancer Inst..

[4] Vallejos C.S., Gomez H.L., Cruz W.R., Pinto J.A., Dyer R.R., Velarde R. (2010). Breast cancer classification according to immunohistochemistry markers: subtypes and association with clinicopathologic variables in a Peruvian hospital database. Clin. Breast Canc..

[5] Sakai T., Kogiso M. (2008). Soy isoflavones and immunity. J. Med. Invest..

[6] Yuseran H., Hartoyo E., Nurseta E.,T., Kalim dan H. (2019). Molecular docking of genistein on estrogen receptors, promoter region of BCLX, caspase-3, ki-67, cyclin D1, and telomere activity. J. Taibah Univ. Med. Sci..

[7] Rajah T.T., Du N., Drews N., Cohn R. (2009). Genistein in the presence of 17-beta-estradiol inhibits proliferation of ER beta breast cancer cells. Pharmacology.

[8] Kang X., Zhang Q., Wang S., Huang X., Jin S. (2010). Effect of soy isoflavones on breast cancer recurrence and death for patients receiving adjuvant. Can. Med. Assoc..

[9] Zhang C., Ho S.C., Lin F., Cheng S., Fu J., Chen Y. (2010). Soy product and isoflavone intake and breast cancer risk defined by hormone receptor status. Off. J. Jpn. Canc..

[10] Thasni K.A.A., Rojini G., Rakesh S.N., Ratheeshkumar T., Babu M.S. (2008). Genistein induces apoptosis in ovarian cancer cells via different molecular pathways depending on breast cancer susceptability gene-1 (BRCA1) status. Eur. J. Pharmacol..

[11] De la Parra C., Castillo-Pichardo L., Cruz-Collazo A., Cubano L., Redis R. (2016). Soy isoflavone genistein-mediated downregulation of miR-155 contributes to the anticancer effects of genistein. Nutr. Canc..

[12] Ye D., Li Z., Wei C. (2017). Genistein inhibits the S-phase kinase-associated protein 2 expression in breast cancer cells. Exp. Ther. Med..

[13] Penner N., Klunk L.J., Prakash C. (2009). Human radiolabeled mass balance studies: objectives, utilities, and limitations. Biopharm Drug Dispos..

[14] Saha G.B. (2010). Fundamentals of Nuclear Pharmacy.

[15] Byczek A., Zawisza-Puchalka J., Gruca A., Papaj K., Grynkiewicz G. (2013). Genistein derivatives regioisomerically substituted at 7-O- and 4′-O- have different effect on the cell cycle. J. Chem..

[16] Spagnuolo C., Russo G.L., Orhan I.E., Habtemariam S., Daglia M. (2015). Genistein and cancer: current status, challenges, and future directions. Adv. Nutr..

[17] Boersen N., Lee T., Hui H.W. (2013). Chapter 4 - Development of Preclinical Formulations for Toxicology Studies. Dalam: *A Comprehensive Guide to Toxicology in Preclinical Drug Development*.

[18] Ramdhani D., Sriyani M.E., Ayu F. (2019). Technetium-99m-labeled genistein as a potential radical scavenging agent. J. Adv. Pharm. Technol. Res..

[19] Souza P.C.T., Textor L.C., Melo D.C., Nascimento A.S., Skaf M.S., Polikarpov I. (2017). An alternative conformation of ERB bound to estradiol reveals H12 in a stable Antagonist position. Sci. Rep..

[20] Eersels J.L.H., Travis M.J., Herscheid J.D.M. (2005). Manufacturing I-123-Labelled radiopharmaceuticals: pitfalls and solutions. J. Label. Compd. Radiopharm..

[21] Kowalsky R.J., Falen S.W. (2004). Radiopharmaceuticals in Nuclear Pharmacy and Nuclear Medicine.

